# Multi-Residue Detection of Eight Glucocorticoids by Nano-Au/Fluticasone Propionate Electrochemical Immunosensor

**DOI:** 10.3390/molecules28186619

**Published:** 2023-09-14

**Authors:** Guozheng Zhao

**Affiliations:** 1 Key Laboratory of Magnetic Molecules & Magnetic Information Materials (Ministry of Education), School of Chemistry and Material Science, College of Food Science, Shanxi Normal University, Taiyuan 030031, China; zhaoguozheng@sxnu.edu.cn; 2 Modern College of Humanities and Sciences of Shanxi Normal University, Linfen 041000, China

**Keywords:** multi-residue, detection, glucocorticoids, electrochemical immunosensor, receptor

## Abstract

Based on an indirect competitive method, a novel nano-Au/fluticasone propionate electrochemical immunosensor was successfully fabricated by combining the nanoscale effect, superior conductivity of nano-Au, stable Au−S chemical bond as well as strong interaction between glucocorticoid and the receptor, which was used to simultaneously detect eight kinds of glucocorticoids. The modified immunosensors’ electrochemical properties were explored by means of a cyclic voltammetry (CV) method and electrochemical impedance spectroscopy (EIS) measurements. Two factors (glucocorticoid receptor concentration, incubation time) were studied in order to obtain the optimal results. The immunosensor presents attractive electrochemical performance with a wide linear range (between 0.1 and 1500 ng⋅mL^−1^) and low detection limit (between 0.057 and 0.357 ng⋅mL^−1^), realizing the rapid multi-residue detection of a large class of glucocorticoids. Two glucocorticoids (hydrocortisone, triamcinolone) were detected in actual skincare samples, which obtained satisfactory detection results.

## 1. Introduction

In 1948, American rheumatologists discovered the miraculous efficacy of hydrocortisone in treating rheumatoid arthritis and won the Nobel Prize [1,2]. Since then, glucocorticoids have become one of the most effective immunosuppressive and anti-inflammatory drugs in clinical practice [3,4]. Glucocorticoids are a class of steroid hormones secreted by the fascicular zone of the adrenal cortex, binding with glucocorticoid receptors to form hormone receptor complexes, which regulate the biosynthesis and metabolism of sugars, fats, and proteins [5,6,7]. Glucocorticoids can inhibit fibroblast proliferation and reduce serotonin formation, thus having a certain whitening effect on the skin [8,9]. Short-term use of skincare cosmetics containing glucocorticoids can create a false appearance of smooth and tender skin [10]. However, high-dose or long-term use may cause various adverse reactions, such as Cushing’s syndrome [11], severe infection [12], osteoporosis [13], diabetes [14], behavioral and cognitive changes, etc. [15,16]. For the sake of monitoring the content of glucocorticoids in skincare cosmetics timely, and to forbid skincare cosmetics containing the excessive glucocorticoids entering the consumer sector, it is crucial to explore novel approaches for rapid multi-residue glucocorticoid detection on site.

Currently, the detection techniques for glucocorticoids include thin-layer chromatography [17], high performance liquid chromatography (HPLC) [18,19], and HPLC tandem mass spectrometry [20,21,22,23]. Thin-layer chromatography is not suitable for current rapid quantitative detection of glucocorticoids. Chromatography is the main detection method for glucocorticoids. However, the detection cost of chromatography is high, sample pretreatment is complex, and a large number of negative samples are repeatedly tested, which is time-consuming and labor-intensive. Chromatographic instruments are expensive, and limited professional testing institutions cannot meet the practical testing needs.

The electrochemical immunosensor is a rapidly developing detection technology in recent years, which uses antigens or antibodies as biological recognition elements and electrochemical detection systems as transducer devices [24,25]. It exhibits the high sensitivity of electrochemical analysis as well as the high specificity of immune reactions, which also has the advantages of low detection equipment cost, simple and fast operation, and is not affected by sample color and turbidity [26]. The immunosensor can directly measure the concentration of the target and is widely used for qualitative and quantitative analysis of various substances in fields such as the environment, food, and clinical diagnosis [27,28,29,30]. Guo et al. proposed an aptamer–antibody sandwich sensor modified by carbon nanotubes for detecting cortisol in human saliva [31]. Pan et al. devised an electrochemical–digital sensor for glucocorticoid hormone detection in human saliva [32]. So far, electrochemical immunosensor detection of glucocorticoids in skincare cosmetics has not been reported in academic research at home and abroad. In this article, using an indirect competitive method, the novel nano-Au/fluticasone propionate immunosensors were prepared by combining nanotechnology, modification of electrode surface and biological immunity to detect simultaneously eight kinds of glucocorticoids, which were applied to the detection of glucocorticoids in actual skincare cosmetics.

## 2. Results and Discussion

### 2.1. Detection Principle of Nano-Au/Fluticasone Propionate Immunosensor

**Scheme 1:**
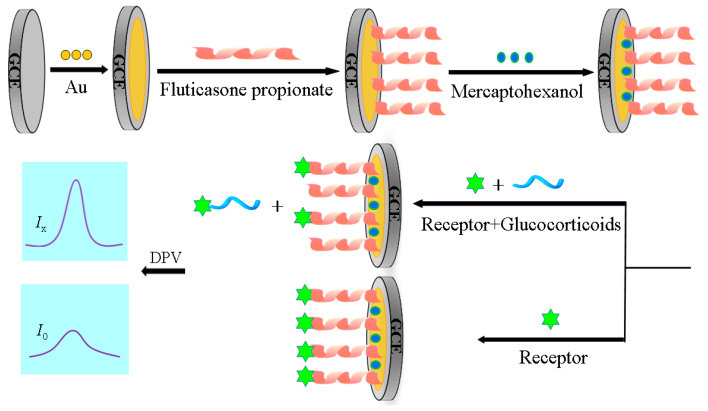
Schematic illustration of nano-Au/fluticasone propionate immunosensor.

The multi-residue detection principle of the nano-Au/fluticasone propionate electrochemical immunosensor was illustrated by Scheme 1. The incubation solution containing the glucocorticoid and receptor were dropped onto the nano-Au/fluticasone propionate immunosensor. Glucocorticoids competed with fluticasone propionate on the nano-Au/fluticasone propionate immunosensor to combine the receptor in the incubation solution. The complex was formed between the glucocorticoid receptor and fluticasone propionate on the immunosensor, resulting in the change of DPV. Furthermore, the binding capacity of the receptor with fluticasone propionate on the immunosensor is inversely proportional with that of glucocorticoid. Accordingly, in a certain range of concentration, it displays a fine linear relationship between the difference of DPV peak current value (Δ*I* = *I*_x_ − *I*_0_, *I*_x_ represents the peak current value corresponding to different concentrations of glucocorticoids, *I*_0_ represents the peak current value corresponding to the blank sample) and the glucocorticoid.

### 2.2. Electrochemical Characterization of Immunosensor

**Figure 1:**
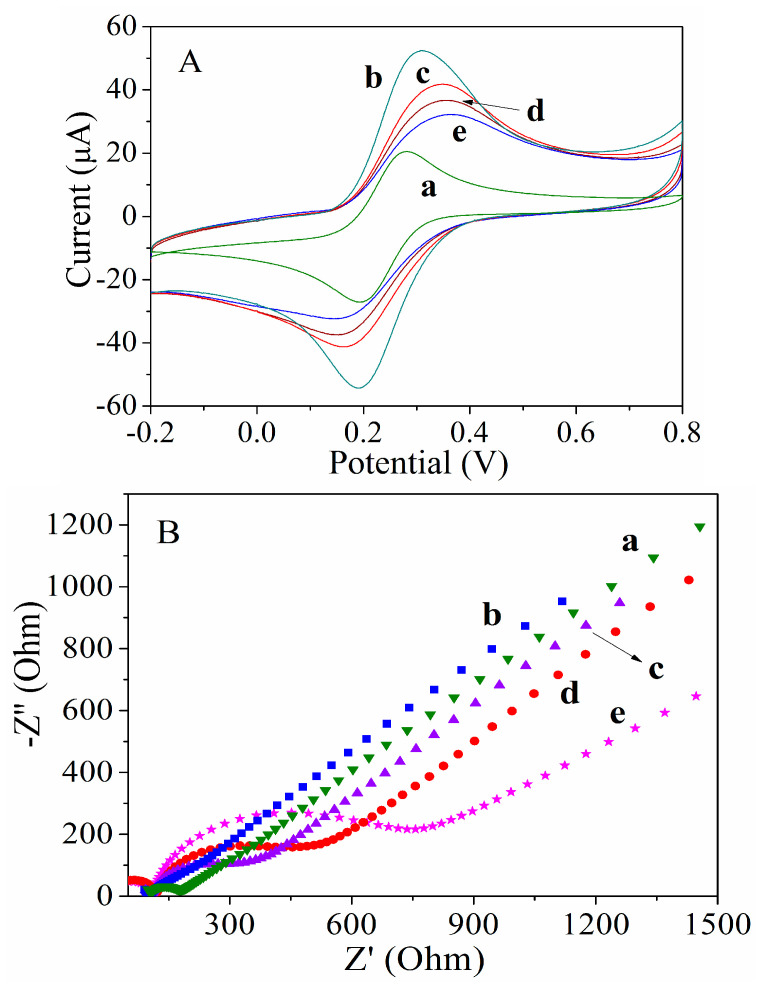
(**A**) CV curves with the modified electrode: (a) GCE; (b) GCE/nano−Au; (c) GCE/nano−Au/fluticasone propionate; (d) GCE/nano−Au/fluticasone propionate/mercaptohexanol; (e) GCE/nano−Au/fluticasone propionate/mercaptohexanol/glucocorticoid receptor. (**B**) EIS curves of modified electrode: (a) GCE; (b) GCE/nano−Au; (c) GCE/nano−Au/fluticasone propionate; (d) GCE/nano−Au/fluticasone propionate/mercaptohexanol; (e) GCE/nano−Au/fluticasone propionate/mercaptohexanol/glucocorticoid receptor.

The CV describes the electrode modification process, showing in Figure 1A. The GCE exhibits a pair of obvious redox peaks (curve a) in PBS solution. Because of excellent conductivity for nano-Au, the electron transfer rate greatly increases, indicating that the peak current for the nano-Au modified electrode (curve b) is significantly stronger than that of the GCE. When fluticasone propionates were modified onto the surface, the curve c decreased, showing that the electrode performance was affected to a certain extent. After closure of the active site by mercaptohexanol, the peak current further decreased, because the steric hindrance generated by mercaptohexanol hindered the electron transfer (curve d). The formation of the complex between fluticasone propionate and receptor results in the peak current value (curve e) being lower than that of GCE/nano-Au/fluticasone propionate/mercaptohexanol, indicating that the receptor has bound to the surface of GCE/nano-Au/fluticasone propionate/mercaptohexanol.

Figure 1B describes the characterization results of EIS for the modified electrode in each stage. According to Figure 1B, due to the excellent charge transfer ability of nano-Au, the electron exchange resistance *R*_et_ typically decreased (curve b) when nano-Au was modified on GCE (curve a), which corresponded well with the results of CV analysis. After applying fluticasone propionate to the nano-Au modified electrode, *R*_et_ increased noticeably (curve c) due to the non-conductivity of small molecules, implying that the influence of molecules on the properties of electrode is considerable. Then, the active site is closed by mercaptohexanol, and *R*_et_ increases significantly (curve d), because the steric hindrance of mercaptohexanol hinders the electron transfer rate. This illustrates the fact that the active sites exist on the modified electrode, which is not occupied by fluticasone propionate. Due to the specific binding of the receptor with fluticasone propionate on the electrode, the steric hindrance is further increased, resulting in a further increase in *R*_et_ (curve e).

### 2.3. Optimization of Condition

**Figure 2:**
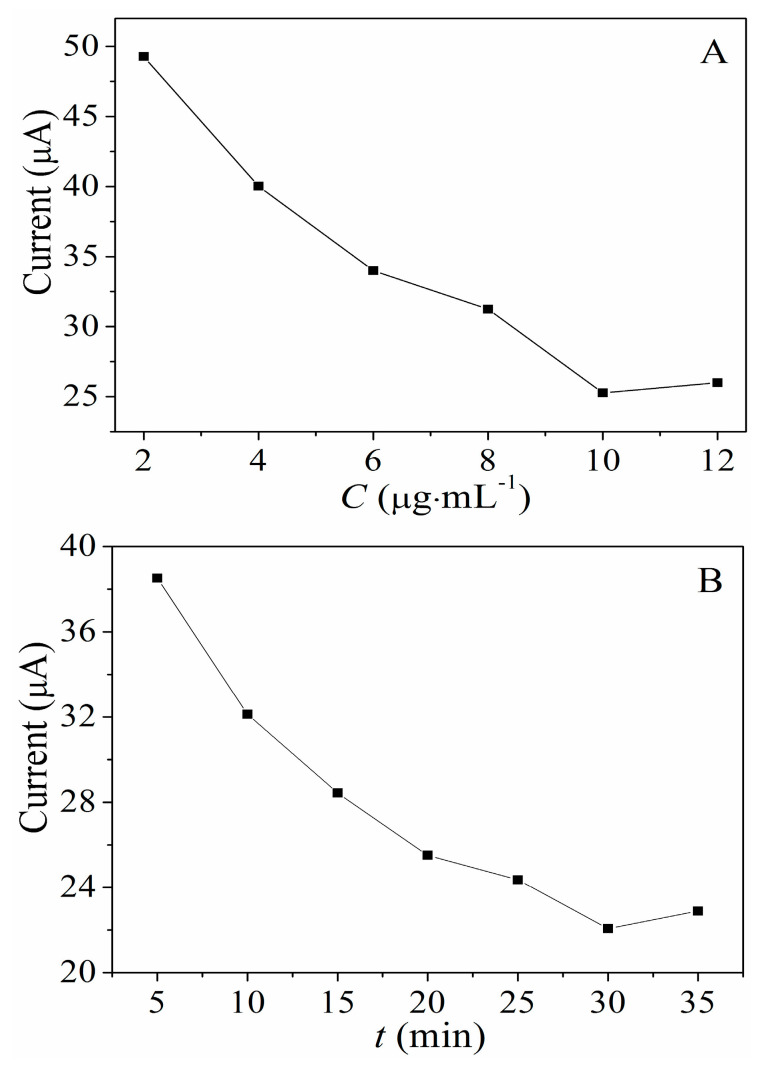
Condition optimization: (**A**) glucocorticoid receptor concentration; (**B**) incubation time.

To achieve the optimal detection effect, the glucocorticoid receptor concentration and incubation time were investigated, shown in Figure 2. According to Figure 2A, as the receptor concentration increased, the peak current of DPV gradually reduced. While the concentration of the glucocorticoid receptor was 10 μg⋅mL^−1^, the peak current reached the minimum. Meanwhile, exceeding 10 μg⋅mL^−1^, there was a slight increase, indicating that the glucocorticoid receptor was saturated. Therefore, 10 μg⋅mL^−1^ was chosen as the appropriate concentration for the receptor.

From Figure 2B, from 5 to 30 min, the binding amount of the receptor with fluticasone propionate increased. At the same time, the peak current of DPV decreased quickly. At 30 min, DPV curve tended to reach its lowest point, then increased, meaning that the binding amount of receptor and fluticasone propionate reached saturation at 30 min. Therefore, 30 min was chosen as the optimal time.

### 2.4. DPV Detection of Glucocorticoids

**Figure 3:**
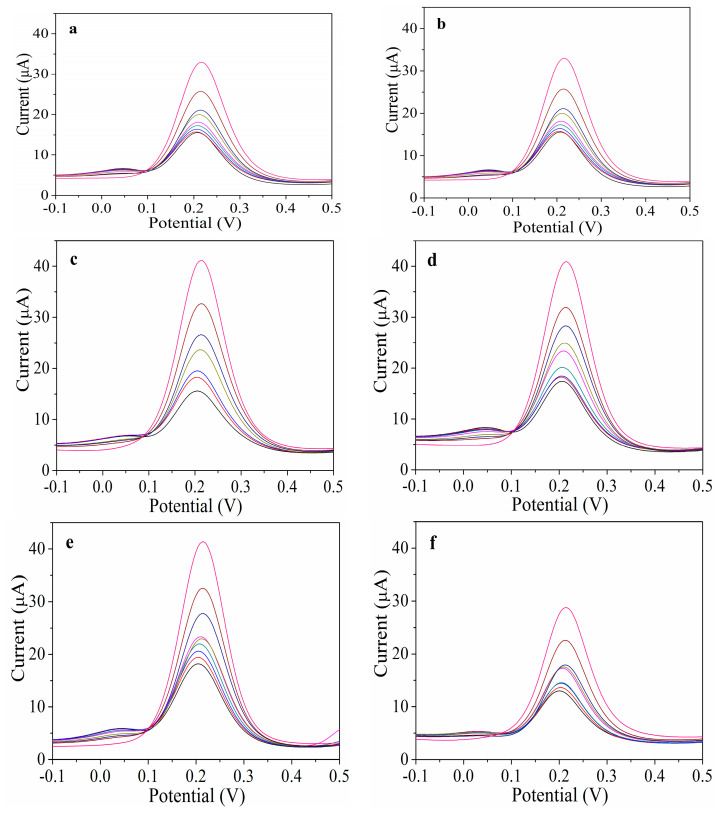

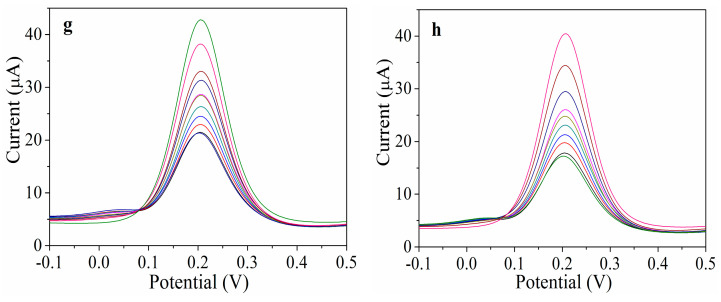
DPV of (**a**) hydrocortisone, (**b**) triamcinolone, (**c**) prednisolone, (**d**) fluticasone propionate, (**e**) triamcinolone acetonide, (**f**) dexamethasone, (**g**) clobetasol 17-propionate, and (**h**) cortisone detection by nano−Au/fluticasone propionate electrochemical immunosensor: from top to bottom, 1500, 1300, 950, 700, 550, 200, 90, 50, 10, 1 and 0 ng·mL^−1^.

Eight kinds of glucocorticoids, including hydrocortisone, triamcinolone, prednisolone, fluticasone propionate, triamcinolone acetonide, dexamethasone, clobetasol 17-propionate, and cortisone were detected by the nano-Au/fluticasone propionate immunosensor. Figure 3 describes the superposition curves of DPV. From Figure 3, when the immunosensor was inserted into the incubation solution containing the glucocorticoid and receptor, the value of the DPV peak current constantly decreased, the reason being that the complex was formed between the glucocorticoid in solution and receptor. The more complex is generated, the more significant the hindrance effect is, and the lower the peak current of DPV. According to the same concentration, the DPV response sequence for the detection of glucocorticoids was clobetasol 17-propionate > triamcinolone acetonide > prednisolone > fluticasone propionate > cortisone > hydrocortisone > triamcinolone > dexamethasone. The higher concentration of glucocorticoids, the larger peak current value of DPV.

Table 1 represents the comparison of detection results of eight glucocorticoids. As demonstrated in Table 1, the dexamethasone and cobetasol 17-propionate exhibit the maximum and minimum slopes, respectively. The correlation coefficients are all higher than 0.9938. The detection limit and linear range for the glucocorticoids are 0.057~0.357 ng⋅mL^−1^ and 0.1~1500 ng⋅mL^−1^, respectively, compared with the previous glucocorticoids detection by different methods, for example, immunochromatographic assay (detection limit 10 ng⋅mL^−1^) [33], UHPLC-Q-Orbitrap HRMS (detection limit 1.0 ng⋅mL^−1^) [34], and SFOD-LPME (detection limit 0.39~0.46 ng⋅mL^−1^) [18], verifying that the nano-Au/fluticasone propionate electrochemical immunosensor displays wonderful detection performance (wider linear range, lower detection limit), ascribing to the nanoscale effect and attractive conductivity of nano-Au. Additionally, it facilitates the immobilization of fluticasone propionate by Au−S chemical bonds and improves a large number of receptors to firmly adsorb on the surface of the modified electrode through strong immunization between receptor and fluticasone propionate.

### 2.5. Stability and Repeatability

The prepared electrochemical immunosensors were placed in a constant temperature drying oven at 37 °C for 1 day, 2 days, 3 days, 4 days, 5 days, 6 days, 7 days, 8 days, 9 days and 10 days, respectively, and then performed multi-residue detection of glucocorticoids. It was found that among the detection results of 10 different electrodes for clobetasol 17-propionate, for the linear relationship’ slope, the standard deviation was 3.6%, indicating that the stability for the electrochemical immunosensor was superior to detect the glucocorticoids. In order to verify the repeatability of the nano-Au/fluticasone propionate immunosensor, three different concentrations of clobetasol 17-propionate were detected by seven different electrodes. The standard deviation of the measured values was 2.8%~6.5%, which indicates that the electrochemical immunosensor can be reused, remarkably increasing the practical application of the immunosensor.

### 2.6. Detection of Actual Samples

Three samples 1 or 2 were chosen (45 ± 0.005 g each) and placed in three beakers. The hydrocortisone/triamcinolone standard solution and extraction solution were added successively to the beakers, which was concentrated to 2 mL, then dried, and an 8 mL ethanol solution (*v*/*v* = 1:1) was added. For investigating the multi-residue detection performance of the nano-Au/fluticasone propionate immunosensor in actual samples, by standard addition recovery experiments, two glucocorticoids (hydrocortisone, triamcinolone) were opted for the detection targets with high, medium and low concentrations. As shown in Table 2 and Table 3, RSD (relative standard deviation) was within 6%. Furthermore, the average recovery was between 92.77%~104.52%, achieving the recovery standard, suggesting that glucocorticoids can be effectively detected by the nano-Au/fluticasone propionate electrochemical immunosensor.

## 3. Experimental Section

### 3.1. Materials and Instruments

Cortisone, hydrocortisone, dexamethasone, triamcinolone, prednisolone, triamcinolone, fluticasone propionate and clobetasol 17-propionate were purchased from Aladdin Biochemical Technology Co., Ltd. (Shanghai, China). Potassium ferricyanide, potassium dihydrogen phosphate and mercaptohexanol were obtained from Sinopharm Chemical Reagent Co., Ltd. (Shanghai, China). Glucocorticoid receptor and two skincare products were bought from Shandong Yishuang Technology Development Co., Ltd. (Jinan, China). and a local supermarket, respectively. The immunosensor’s electrochemical performance was measured by a CHI600E electrochemical workstation, which was purchased from Chenhua Apparatus Co., Ltd. (Shanghai, China).

### 3.2. Preparation of Nano-Au/Fluticasone Propionate Immunosensor

A glass carbon electrode (GCE) with a diameter of 3 mm was used for the preparation of the immunosensor. The GCE was cleaned before each experiment. The pretreated GCE was inserted into a 100 mg⋅mL^−1^ chloroauric acid solution. The nano-Au was covered on the GCE by the electrochemical precipitation method (voltage: −0.2 V, deposition time: 60 s). This modified electrode was washed and dried at 20 °C. Next, 15 μL 0.1 M fluticasone propionate was dropped onto the surface of the modified electrode, which was incubated at 37 °C for 2 h. The modified electrode was incubated in 100 mg⋅mL^−1^ of mercaptohexanol for 40 min in order to block the active sites. Eventually, the nano-Au/fluticasone propionate immunosensor was constructed by washing the electrode with a PBS solution.

### 3.3. Characterization

The electrochemical properties were characterized through CV and EIS, including GCE, GCE/nano-Au, GCE/nano-Au/fluticasone propionate, GCE/nano-Au/fluticasone propionate/mercaptohexanol, GCE/nano-Au/fluticasone propionate/mercaptohexanol/glucocorticoid receptor. The CV diagram was evaluated with a potential scanning range (between −0.2 and 0.8 V) and scanning rate (100 mv⋅s^−1^). The EIS curve was measured with the scanning frequency (between 0.1 and 10^5^ Hz) and pulse amplitude (5 mV) in a 0.1 M KCl and 5 mM K_3_[Fe(CN)_6_] solution.

### 3.4. Optimization of Condition

The glucocorticoid receptor concentration and incubation time were explored to detect glucocorticoids by a nano-Au/fluticasone propionate electrochemical immunosensor under optimal conditions at 37 °C.

Optimizing the glucocorticoid receptor concentration in incubation solution: Different concentrations of glucocorticoid receptor in PBS incubation solution were prepared and coated on nano-Au/fluticasone propionate electrochemical immunosensors. After incubation at 37 °C for 40 min, the nano-Au/fluticasone propionate-modified electrode was washed three times and analyzed by DPV (differential pulse voltammetry), which was determined by a three-electrode system on an electrochemical workstation.

Optimizing the incubation time: A 15 μL mixed incubation solution (glucocorticoid receptor and PBS) was coated on the nano-Au/fluticasone propionate electrochemical immunosensor. The DPV was monitored every 5 min.

### 3.5. Glucocorticoids Detection

To investigate multi-residue detection properties, the nano-Au/fluticasone propionate electrochemical immunosensors were applied to the quantitative DPV detection of eight glucocorticoids standard solutions. Based on the optimal glucocorticoid receptor concentration and incubation time, different concentrations of glucocorticoid solutions (15 μL, the ratio of glucocorticoid receptor to PBS is 4:1) were dropped onto the nano-Au/fluticasone propionate immunosensors. After incubation, the nano-Au/fluticasone propionate electrochemical immunosensor was washed by PBS and put it into a PBS buffer solution (0.1 M, pH 7.4) of 2 mM K_3_[Fe(CN)_6_]/K_2_[Fe(CN)_6_]. The DPV diagram was evaluated with the potential scanning range (between -0.2 and 0.5 V) and pulse amplitude (50 mV).

## 4. Conclusions

In this work, the rapid multi-residue detection of eight kinds of glucocorticoids with an indirect competitive immunoassay was achieved by the prepared nano-Au/fluticasone propionate electrochemical immunosensor on the basis of the nanoscale effect, superior conductivity of nano-Au, strong Au−S bond and specific immune effects between the glucocorticoid and receptor. During the incubation process, the optimal glucocorticoid receptor concentration and incubation time are 10 μg⋅mL^−1^ and 30 min, respectively. The peak current of DPV decreases with the decrease in the concentration of glucocorticoids. This is because that the complex is formed between glucocorticoid and receptor, hindering the transfer of electrons on the modified electrode surface. The nano-Au/fluticasone propionate immunosensor shows favorable performance (wider linear range: 0.1~1500 ng⋅mL^−1^; lower detection limit: 0.057~0.357 ng⋅mL^−1^; fine correlation coefficient: 0.9938~0.9967) as well as preferable stability and repeatability, which can be attributed to the novel design concept. The average recovery and RSD of hydrocortisone/triamcinolone detection in actual skincare samples were carried out, suggesting considerable potential in multi-residue detection of environmental and cosmetic safety.

## Figures and Tables

**Table 1:** Detection results of eight glucocorticoids.

Detection Object	Linear Relationship	Correlation Coefficient	Linear Range (ng⋅mL^−1^)	Detection Limit (ng⋅mL^−1^)
Fluticasone propionate	Y = 0.0161X + 11.2106	R = 0.9938	0.1~1500	0.221
Hydrocortisone	Y = 0.0157X + 18.3992	R = 0.9955		0.072
Triamcinolone acetonide	Y = 0.0160X + 17.9102	R = 0.9946		0.296
Cobetasol 17-propionate	Y = 0.0155X + 15.5987	R = 0.9957		0.156
Prednisolone	Y = 0.0164X + 14.2092	R = 0.9954		0.357
Cortisone	Y = 0.0171X + 13.3632	R = 0.9967		0.091
Dexamethasone	Y = 0.0173X + 12.2203	R = 0.9963		0.057
Triamcinolone	Y = 0.0167X + 16.5602	R = 0.9952		0.278

**Table 2:** Detection of hydrocortisone in skincare sample 1.

Amount Added (ng⋅mL^−1^)	Actual Concentration (ng⋅mL^−1^)	Recovery (%)	Average Recovery (%)	RSD (%)
100	95.78	95.78	92.77	3.32
	89.63	89.63		
	92.91	92.91		
400	409.66	102.42	102.00	2.56
	417.53	104.38		
	396.78	99.20		
1000	936.82	93.68	94.20	3.66
	978.71	97.87		
	910.40	91.04		

**Table 3:** Detection of triamcinolone in skincare sample 2.

Amount Added (ng⋅mL^−1^)	Actual Concentration (ng⋅mL^−1^)	Recovery (%)	Average Recovery (%)	RSD (%)
100	110.39	110.39	103.88	5.70
	102.45	102.45		
	98.80	98.80		
400	407.12	101.78	104.52	2.31
	425.43	106.36		
	421.67	105.42		
1000	950.32	95.03	93.08	2.13
	931.50	93.15		
	910.70	91.07		

## Data Availability

All datasets generated for this study are included in the article.
